# Improvement of the Bag-Mediated Filtration System for Sampling Wastewater and Wastewater-Impacted Waters

**DOI:** 10.1007/s12560-017-9311-7

**Published:** 2017-07-03

**Authors:** Christine Susan Fagnant, Liliana Margarita Sánchez-Gonzalez, Nicolette A. Zhou, Jill Christin Falman, Michael Eisenstein, Dylan Guelig, Byron Ockerman, Yifei Guan, Alexandra Lynn Kossik, Yarrow S. Linden, Nicola Koren Beck, Robyn Wilmouth, Evans Komen, Benlick Mwangi, James Nyangao, Jeffry H. Shirai, Igor Novosselov, Peter Borus, David S. Boyle, John Scott Meschke

**Affiliations:** 1 Department of Environmental and Occupational Health Sciences, University of Washington, 4225 Roosevelt Way NE, Suite 100, Seattle, WA 98195 USA; 2 PATH, 2201 Westlake Ave., Suite 200, Seattle, WA 98121 USA; 3 Department of Mechanical Engineering, University of Washington, Stevens Way, Box 352600, Seattle, WA 98195 USA; 4 Kenya Medical Research Institute, Center for Virus Research, Mbagathi Road, P.O. Box 54628, Nairobi, 00200 Kenya

**Keywords:** Environmental surveillance, Poliovirus, Enterovirus, Pathogens, BMFS, Wastewater

## Abstract

Environmental surveillance of poliovirus (PV) plays an important role in the global program for eradication of wild PV. The bag-mediated filtration system (BMFS) was first developed in 2014 and enhances PV surveillance when compared to the two-phase grab method currently recommended by the World Health Organization (WHO). In this study, the BMFS design was improved and tested for its usability in wastewater and wastewater-impacted surface waters in Nairobi, Kenya. Modifications made to the BMFS included the size, color, and shape of the collection bags, the filter housing used, and the device used to elute the samples from the filters. The modified BMFS concentrated 3–10 L down to 10 mL, which resulted in an effective volume assayed (900–3000 mL) that was 6–20 times greater than the effective volume assayed for samples processed by the WHO algorithm (150 mL). The system developed allows for sampling and in-field virus concentration, followed by transportation of the filter for further analysis with simpler logistics than the current methods. This may ultimately reduce the likelihood of false-negative samples by increasing the effective volume assayed compared to samples processed by the WHO algorithm, making the BMFS a valuable sampling system for wastewater and wastewater-impacted surface waters.

## Introduction

Environmental surveillance is crucial for monitoring and preventing the spread of pathogens, which are present in wastewater, surface waters impacted by wastewater, recreational waters, and drinking water sources (Betancourt et al. 2014; Deboosere et al. 2011; Fuhrimann et al. 2015) that humans and animals regularly contact. Environmental surveillance can highlight epidemiological patterns, especially for diseases with high subclinical infection rates, where pathogens silently circulate. For example, although less than 1 in 100 poliovirus infections results in acute flaccid paralysis, the virus is shed in the stool of all infected persons (Nathanson and Kew 2010). Environmental surveillance of wastewater can detect poliovirus from these asymptomatic carriers, resulting in early pathogen detection, focused vaccine efforts, and eventual eradication certification (Hovi et al. 2012).

As pathogens are typically present in low numbers in wastewater-impacted surface waters, detection can be challenging. The likelihood of detection can be improved through concentration of the environmental samples, which results in an increase in the effective volume assayed. Typically, after grab sample collection, concentration occurs in the laboratory by chemical and/or physical mechanisms. For example, when sampling for poliovirus from wastewater and wastewater-impacted surface waters, the two-phase grab sample method commonly used by the World Health Organization (WHO) involves concentrating 500 mL down to 10 mL by a two-phase separation method using polyethylene glycol (PEG) and dextran (World Health Organization 2015). For physical concentration, vacuum-driven or pump-driven filtration is often used (Gantzer et al. 1997; Karim et al. 2009; Katayama et al. 2002). These types of filtration methods require concentration to be completed in the laboratory, which requires transportation and storage of samples prior to processing. In low-resource settings, such sampling methodology can be expensive and logistically challenging to perform.

On-site filtration could be used to concentrate large sample volumes, resulting in a high effective volume assayed and an increased likelihood of pathogen detection. Filtration at the field site is advantageous because it reduces the need to transport large volumes of potentially biohazardous materials, allows for sampling of larger volumes without requiring transportation or shipment of heavy samples, and reduces the storage footprint in space-limited laboratories. However, in-field filtration typically requires the use of an electric-powered pump (Corsi et al. 2014). This can make filtration in the field prohibitive as pumps are expensive, require a continual power source, and are large and heavy. Portable, low-cost considerations for in-field filtration are needed.

**Fig. 1:**
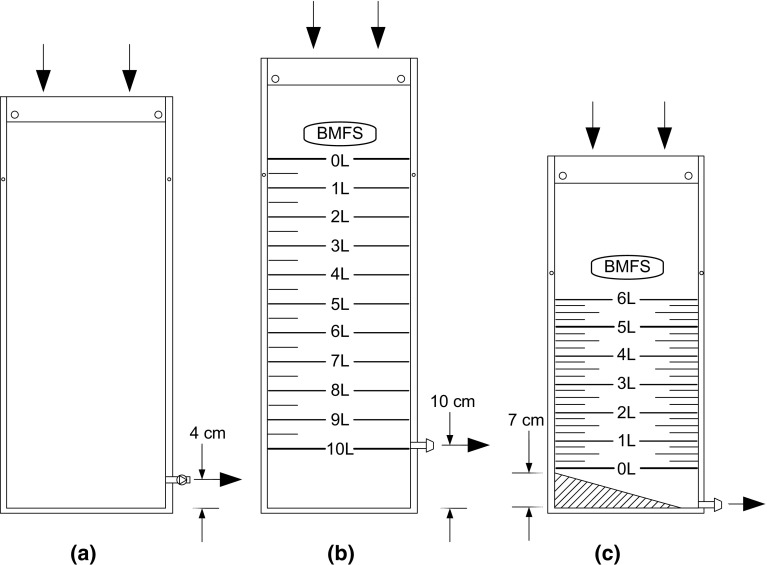
Schematic of collection bag. **a** First-generation *yellow*, 10-L bag. Bag outlet was located 4 cm above the bag’s bottom. **b** Second-generation drab *green*, 10-L bag. Bag outlet was located 10 cm above the bag’s bottom. 0.5-L volume increments were demarcated on the bag. **c** Third-generation drab *green*, 6-L bag. Bag outlet was located at the bag’s bottom, and a 15.5° slope was heat sealed onto the bottom of the bag. 0.25-L volume increments were demarcated on the bag

The objective of this study was to improve the usability and sensitivity of the previously developed bag-mediated filtration system (BMFS) (Fig. 1) (Fagnant et al. 2014). This system enables in-field filtration of samples while requiring no power. It is compact enough to fit inside of a backpack and is simple to use. In previous tests, the BMFS could filter more than 10 L of effluent from primary clarifiers at a local wastewater treatment plant and 3–5 L of influent wastewater (Fagnant et al. 2014), which increased the virus concentration compared to the recommended two-phase sampling method by the WHO for poliovirus detection. Filtration of wastewater and wastewater-impacted surface waters in Nairobi, Kenya demonstrated the usability of the BMFS, and deployment feedback-guided modifications to the BMFS design. This paper describes those modifications.

## Materials and Methods

### BMFS Design

The basic BMFS consists of a 6- to 12-L collection bag, filter, collapsible tripod stand, personal protective equipment, and decontamination supplies. All supplies are transportable in an insulated backpack.

Sample collection bags underwent three design iterations (Fig. 1; Table 1). First-generation collection bags held 10-L, bright yellow polyurethane-coated nylon sock bags with 1-cm heat-sealed seams (Fig. 1a). The material was flexible, durable, and water-tight. Samples were collected at the top, which was held open by a detachable brass ring that was inserted through holes at the bag’s top opening. A rope was attached to the ring for sample collection. Samples flowed through a quick-disconnect check valve port located 4 cm above the bag’s bottom, to which the filter was attached after collection. Reinforced holes on the bag’s side seam allowed it to hang on the tripod stand (Fagnant et al. 2014).

**Table 1:** Modifications to the bag-mediated filtration system kit

Variable	Version	Modification	Justification
Pre-screen mesh	1	30 × 76 cm (10 L)	
	2, 3	Pre-screen mesh	A pre-screen mesh on the collection bag inlet prevented coarse sediment and debris from entering the bag
Collar	1	Detachable brass ring	
	2, 3	Stainless steel rolled ring clamp	The stainless steel rolled ring clamp held the pre-screen mesh onto the collection bag inlet The clamp had a side loop for rope attachment The clamp had a second side angled loop that facilitated easy placement of the filled collection bag on the tripod stand
Sample collection bag Outlet port	1	Quick-disconnect check valve port Located 4 cm above the bag’s bottom	
	2	Open ¼-inch barbed tubing adapter port Located 10 cm above the bag’s bottom	Due to clogging in the first-generation bag, a straight barbed tubing adapter replaced the quick-disconnect check valve The catchment area below the outlet valve increased from 4 to 10 cm for capture of settled solids. Settleable solids accumulated below the outlet prior to filtration, thus minimizing solids entering and clogging the filter
	3	Open ¼-inch barbed tubing adapter port Located at the bottom of the bag Heat-sealed 15.5° slope angles toward the outlet	The lower sediment catchment area of the bag was ineffective as settled solids resuspended during the rolling of the bag when pressure was applied, released, and reapplied. Therefore, the catchment area was removed A 15.5° slope was heat sealed onto the bottom of the bag. This enabled bleeding of settled solids to a second vessel prior to filtration of the remaining clarified sample
Sample collection bag Size	1	30 cm × 76 cm (10 L)	
	2	29 cm × 81 cm (10 L)	The bag height increased due to the switch from the detachable brass ring to the stainless steel rolled ring clamp. The top of the bag folds over the metal clamp for attachment, requiring a greater bag height
	3	28 cm × 64 cm (6 L) 28 cm × 94 cm (12 L)	Typically, field technicians processed less than 4-L wastewater or wastewater-impacted surface waters in Nairobi, making collection of a 10-L water sample unnecessary. A 6-L bag enabled bleeding and collection of an initial 1 L settled volume, and filtration of the remaining clarified 5 L A 12-L bag facilitated use of a new protocol (not discussed in this work). The large bag enabled bleeding and collection of an initial 2 L settled volume, and filtration of the remaining clarified 10 L through two filters
Sample collection bag Color Volume markings	1	Bright yellow	
	2	Drab green with white screen printing Volumetric demarcations: 0.5-L increments	Bright yellow bags attracted attention from on-lookers. Therefore, the color was changed to drab green to reduce attention Volumetric demarcations were added, enabling measurement of the filtered volume
	3	Drab green with white screen printing Volumetric demarcations: 0.25-L increments	The volumetric demarcations were changed to 0.25-L gradations to facilitate easier and more accurate measurement of the filtered volume

Second-generation collection bags held 10 L, were drab green with white screen printing that demarcated 0.5-L volume increments, and had 1-cm heat-sealed seams (Fig. 1b). Samples were collected at the top, which was held open by a stainless steel rolled ring clamp with a spring form mechanism. The inner and outer rings were attached by a lanyard. The clamp held a pre-screen mesh on the inlet of the bag, and the mesh had a uniform pore size. Several different mesh pore sizes were tested, ranging from 249 µm to 1.2 mm, and a final pore size of 249 µm was selected. The clamp also included a loop for rope attachment and an angled loop for easy placement on the tripod stand. Samples flowed through an open ¼-inch-diameter barbed tubing adapter port located 10 cm above the bag’s bottom. An 8-cm section of tubing with a tubing clamp was attached to the port, which was clamped while the bag was filling.

Third-generation collection bags held 6 or 12 L, were drab green with white screen printing that demarcated 0.25-L volume increments, and had 1-cm heat-sealed seams (Fig. 1c). Sample collection was completed the same as for the second-generation bag with the clamp and pre-screen mesh. The port was moved to the bottom of the bag and a 15.5° slope was heat sealed onto the bottom of the bag to angle sediments toward the outlet port so they could be collected prior to filtration.

### Sample Collection and Filtration

#### Laboratory Testing of Filters

For laboratory testing, 10 L influent wastewater (after bar screens) grab samples were collected from the West Point Wastewater Treatment Plant in Seattle, WA, USA. Samples were collected by bucket and rope, poured into 20-L carboys, and transported back to the laboratory where they were stored at room temperature until processing (within 7 days of collection). Samples were subsequently filtered through 5-cm ViroCap™ filters (Scientific Methods, Inc., Granger, IN, USA) using a peristaltic pump.

#### Field Testing of BMFS

For field testing, 10 L surface water grab samples were collected from Lake Union in Seattle, WA, USA and from four study sites in Nairobi, Kenya. In Nairobi, samples were collected from the Motoine River (which is impacted by untreated latrine waste) bordering the Kibera informal settlement, a latrine waste stream in the Mathare informal settlement that flows to the Mathare River, and sewer conveyance lines in the Eastleigh neighborhood (two sites). These collection sites will be referred to as Kibera, Mathare, Eastleigh A, and Eastleigh B.

**Fig. 2:**
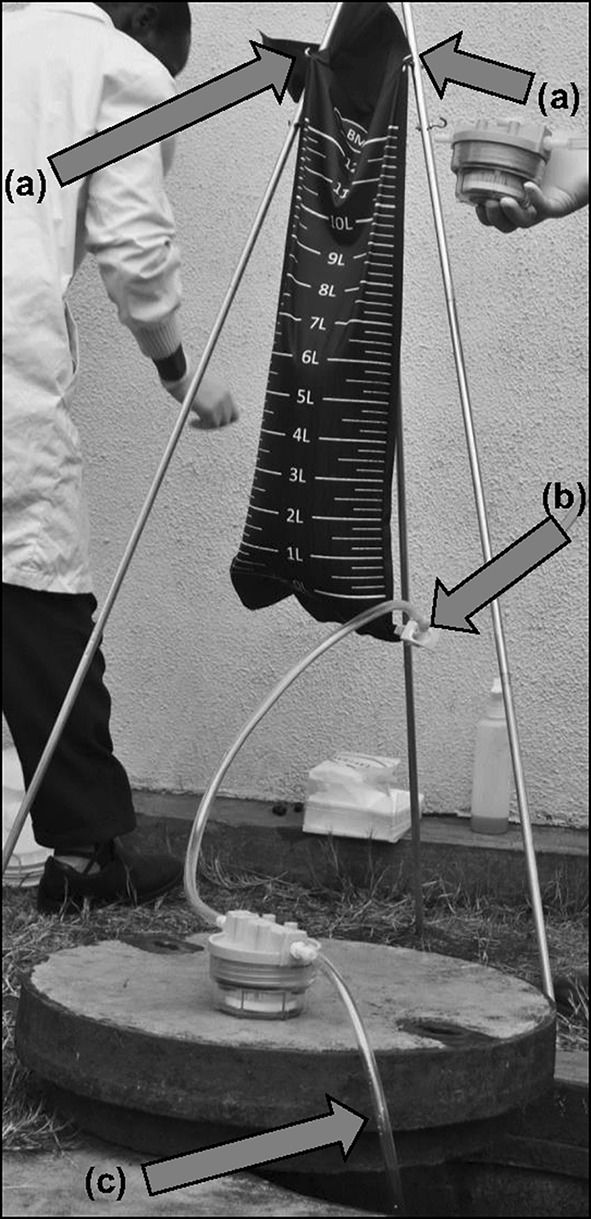
BMFS sample filtration. **a** Collection bag hangs on tripod stand by hooks and holes in the side seams; **b** after the collection bag is hung, the ViroCap filter attaches via a tubing adapter; **c** ViroCap filtrate drains to the source water

For sample collection, the BMFS collection bag was lowered into the source by the rope and filled, then hauled up and hung on the tripod. The sample settled for 15 min, and then the settled solids and debris were drained until 1 L of the sample was drained or until a clarified liquid passed from the bag. Tubing and a cartridge filter were subsequently attached to the outlet port (Fig. 2). The tubing clamp was opened and the sample filtered under gravity, with a 0.5-m head for disposable filter housings and a 0.7-m head for reusable filter housings. When the gravity head was inadequate to drive filtration, the collection bag was sealed with a clamp, removed from the tripod stand, and rolled by hand to apply pressure and filter the remaining sample. Samples were filtered through 5-cm ViroCap filters up to 10 L or until clogging. Filters were returned to the laboratory under cold chain and processed.

### ViroCap Filter Housings

Commercially produced, disposable ViroCap filter housings and reusable ViroCap filter housings designed for this study were used for laboratory tests and field sampling. The reusable housings aimed to reduce the system’s environmental footprint and reduce the holdup/elution volume from 175 to 100 mL. The holdup volume is defined as the non-solid void within the filter housing, a measurement used in calculating the concentration factor of the system. The reusable housing sumps were custom-designed, injection-molded, made from clear polycarbonate (Protolabs, Maple Plain, MN, USA), and used in conjunction with a reinforced polypropylene lid (Pentek, Inc, Upper Saddle River, NJ, USA). The filter is seated in the body of the housing and the lid is tightened with the use of a customized wrench to secure the filter in place. An O-ring sits at the interface between the housing and lid to provide a water-tight, low-pressure seal. The threaded lid of the housing allows for an expandable seal to accommodate a wide range of filter heights, from 49.784 to 51.816 mm (1.96–2.04 inches). An elastomer O-ring seal prevented leakage. Polypropylene barbed tubing adapters (1/4 NPT × 0.9525 cm inner diameter) connect the housing and collection bag. All housing components can withstand autoclaving (121 °C, 124 kPa, 30 min) for at least five cycles with no loss of sealing integrity (data not shown). They can also be disinfected by soaking for 30 min in a Virkon™ (DuPont, Wilmington, DE, USA) or 0.5% HOCl (bleach) solution.

### Elution and Secondary Concentration

ViroCap filters were eluted in a 1.5% beef extract and 0.05 M glycine solution, at pH 9.5. The eluent was poured into the filter housing inlet, let stand for a specified period of time, and then pumped out with a peristaltic pump. The eluate was adjusted to pH 7.0–7.5 with 5 M HCl and 5 M NaOH. Disposable housings were eluted with 175 mL and the eluent was held in the filter housing for 30 min. Reusable housings were eluted by two methods: (1) a single elution in which they were eluted with 100 mL and the eluent was held in the filter housing for 30 min, and (2) a double elution in which they were eluted two times with 100 mL and the eluent was held in the filter housing for 15 min for each elution.

Secondary concentration was performed by PEG/NaCl precipitation. PEG 8000 (14 g/100 mL) and NaCl (1.17 g/100 mL) were added to the sample and dissolved by vigorous shaking (5 min). Samples were shaken overnight (200 RPM, 4 °C) and then centrifuged (6000×*g*, 4 °C, 30 min). The supernatant was discarded and the pellet was resuspended in 10 mL of phosphate-buffered saline.

### Elution Device

**Fig. 3:**
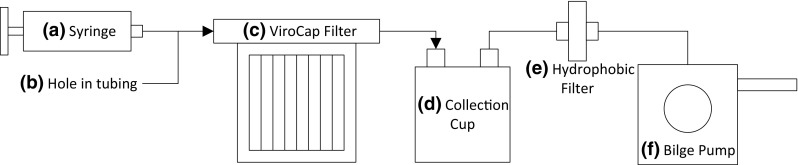
Schematic of elution device. **a** Syringe to inject the eluent; **b** hole in injection tubing allows air flow during eluent injection and prevents liquid lock; **c** ViroCap filter receives the eluent and viruses adsorbed to the filter are released to the eluate; **d** collection cup encloses the eluate during final collection, reducing the potential for cross-contamination by aerosolization of pathogens from popping bubbles; **e** hydrophobic filter prevents aerosols from entering the bilge pump; **f** manual bilge pump drives the eluate movement from the ViroCap filter to the collection cup by vacuum pressure

An elution apparatus was designed to eliminate the need for a peristaltic pump during elution (Fig. 3). A disposable syringe (Fig. 3a) injects 100 mL of eluent into the reusable filter housing. A vent was placed in the injection line to allow air in the injection line to escape, thus preventing the formation of air lock in the line and the filter housing (Fig. 3b). The eluate is held in the ViroCap filter (Fig. 3c) for 30 min until it is eluted through the filter and transferred into a collection container (stool collection cup) with a modified lid (Fig. 3d). An Alfa Marine manual bilge pump provides suction for the sample elution (Fig. 3f) (Alfa Marine Co., Ltd, Shanghai, China). A Whatman^®^ Vacu-Guard hydrophobic filter (Fig. 3e; GE Healthcare Bio-Sciences, Pittsburgh, PA, USA) is placed on the collection cup outlet to prevent liquid from entering the pump.

### Water Sample Characteristics

Concentrations of total dissolved solids (TDS) were determined for the water samples collected at the Kibera, Mathare, Eastleigh A, and Eastleigh B sites. TDS were determined by Kenya Standard Method 459 (Gazette 1985).

### Filter Clogging Challenge

To determine particle sizes that resulted in filter clogging in the BMFS, 10 L of tap water seeded with 25 g soil particles was filtered through the BMFS in the laboratory. Particle sizes included <850, <180, <150, <63, and <25 µm. Filters were also tested for clogging by filtering 10 L of surface water and influent wastewater samples from Seattle, WA, USA. Field testing was conducted at the Kibera, Mathare, Eastleigh A, and Eastleigh B sites in Nairobi.

### Poliovirus Culture and Recovery Experiments

The vaccine strain of poliovirus type 1 (PV1) stocks were prepared by confluent lysis of buffalo green monkey kidney (BGMK) cell monolayers (Sobsey and Jones 1979). PV1 was extracted using Vertrel XF (E. I. du Pont de Nemours and Company, Wilmington, DE, USA) and purified stocks were stored at −80 °C (Mendez et al. 2000).

To determine the poliovirus recovery efficiency using the modified BMFS, laboratory-seeded experiments were completed. Influent wastewater samples were seeded with ~10 PFU/mL PV1 prior to filtration through ViroCap filters in either disposable or reusable housings. Samples were then eluted using a single elution (175 mL) with the disposable housing, and either a single elution (100 mL) or a double elution (100 mL each) with the reusable housing. Samples were then concentrated to 10 mL by PEG/NaCl and analyzed for PV1 by plaque assay. Viruses were enumerated on 95% confluent BGMK cells as previously described and modified to include an Avicel RC-581 (FMC Corporation, Philadelphia, PA, USA) overlay (Sobsey and Jones 1979; Matrosovich et al. 2006). Assays were performed in triplicate onto 9.5 cm^2^ wells using 200-µL aliquots of relevant dilutions. Infected cells were incubated at 37 °C and 5% CO_2_ for 40–44 h and then stained with 2% crystal violet in 20% methanol. Plaques were counted for infectious virus enumeration. Viral recovery was calculated by dividing the recovered viral count by the seeded viral count.

## Statistical Analyses

The confidence intervals (CI) on sample volume and filtration time were calculated by

1 $$ 95\% {\text{CI}} = \bar{x} \pm \frac{t \times \sigma }{\sqrt n } $$

where $\bar{x}$ is the mean, t is the critical value when the degrees of freedom is (*n* − 1) and *α* is 0.05, *σ* is the standard deviation, and *n* is the number of samples.

Unpaired Student’s or Welch’s *t* tests were used to compare PV recoveries between filtration through a disposable versus reusable filter housing, while using a single or double elution.

## Results and Discussion

### Modifications of the BMFS Design

#### Collection Bag Design

The design of the BMFS went through several iterations based on results from field tests concerning the filterability of the samples and other observations (Table 1; Fig. 1).

For the first generation of the BMFS, laboratory experiments suggested that collection and processing up to 10 L of primary or secondary wastewater samples was feasible when using the ViroCap filter (Fagnant et al. 2014). During field evaluations, however, high amounts of sediments clogged the system after filtration of 2–3 L of wastewater and wastewater-impacted surface waters from several sites in Nairobi. The clogging occurred in either the ViroCap filter (Kibera site) or collection bag (Eastleigh B site). It was thought that the clogging of the filter was due to fine suspended particles in the water samples. Clogging of the collection bag likely occurred inside of the outlet check valve due to an accumulation of celluloid material observed in the water, which prevented liquid from exiting the bag.

Based on these observations, the BMFS was modified to create a second-generation form (Table 1; Fig. 1b). Laboratory evaluation demonstrated that collection and filtration of 10 L of influent wastewater was possible with the ViroCap filters, though filter clogging issues remained during the field evaluation. Collection bag clogging was not encountered in the field because the check valve choke point was removed (Table 1). Water analyses at the four sites in Nairobi found that TDS concentrations were heavily loaded in Mathare and Eastleigh A, and moderately loaded in Kibera and Eastleigh B (Table 2). A visual examination of the sampling process at Kibera revealed visible particle loading of the 249-µm pre-screen mesh and a significant amount of fine sediments passing through the pre-screen. At all sites, the filters clogged after approximately 3–4 L of sample water was filtered. It is likely that filters were clogging during the field evaluation due to fine suspended particles or other constituents blocking the filter sites (e.g., fats, soap). This observation was supported by laboratory experiments, which found that fine particles (<25 µm) clogged the ViroCap filter more than twice as quickly as with larger particles (<63 µm). Filters loaded with particles <850, <180, <150, and <63 µm in size experienced no clogging issues for gravity filtration of 10 L of tap water with 2500 mg/L particle matter.

**Table 2:** Water constituents for sampling sites in Nairobi

Site	Total dissolved solids (mg/L)
Kibera	483
Mathare	805
Eastleigh A	805
Eastleigh B	511
Typical medium strength wastewater^a^	500

One sample was evaluated for each site

^a^(Tchobanoglous et al. 2003)

The third generation of the BMFS collection bag was modified based on these new observations (Table 1; Fig. 1c). This new design enabled a 15-min clarification/settling period, followed by bleeding of the settled solids to a second vessel prior to filtration of the remaining clarified sample. Initial settling occurred while the user performed other tasks; typically, 15 min passed while the bag hung on the tripod stand. After settling, 0.5–1.0 L of settled sediments were collected in a Whirl–Pak^®^ bag (Nasco, Fort Atkinson, WI, USA), and the clarified sample was filtered until the filter clogged. If the full clarified volume was filtered, the collected sediments were poured back into the sample collection bag for filtration.

#### Filter Design

Disposable ViroCap filter housings were used during the initial development of the BMFS. The filter design was modified based on observations from field tests that indicated slow filtration. Reusable housings were designed to reduce the system’s environmental footprint and reduce the holdup volume from 175 to 100 mL.

Reduction of the holdup/elution volume from 175 mL with the disposable housing to 100 mL with the reusable housing improves the likelihood of pathogen detection. This is due to an increase in the concentration factor from 1:57 for the disposable housing to 1:100 for the reusable housing when a 10-L sample is filtered. Therefore, if the eluate is assayed directly, the effective volume assayed and subsequent likelihood of detection is increased by 75% for the reusable housing over the disposable housing. A smaller eluate volume also reduces the processing time in the laboratory required for secondary concentration. Secondary concentration may be desirable to increase the effective volume assayed for viruses expected to be present in low concentration or for assays requiring low sample volumes.

**Fig. 4:**
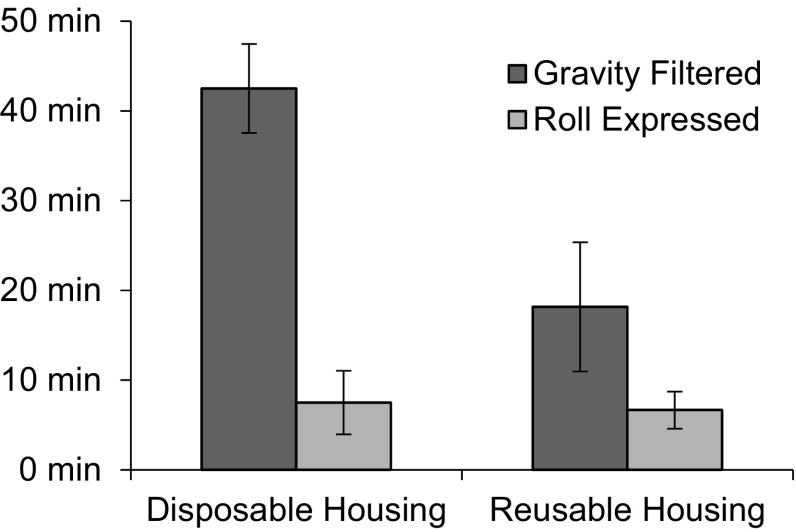
Time required to filter samples at the Kibera site in Nairobi. Total volume filtered ranged from 2.9 to 4.0 L. *n* = 2 for disposable housing, *n* = 6 for reusable housing. *Error bars* represent standard deviation

The disposable and reusable ViroCap filter housings were evaluated for use with the BMFS and their performance was measured by comparing the time required for gravity filtration (passive) and by roll-expressed filtration (active) (Fig. 4). Similar times were required for active filtration of 2.5 to 4 L wastewater-impacted surface water when using the disposable housings and the reusable housings. By contrast, the disposable housings required 20 min longer on average during passive filtration. Passive filtration provided 4.8 kPa pressure on the system for the disposable housing and 6.9 kPa for the reusable housing. Active filtration exhibited an average of 27.6 kPa on the system and up to 44.8 kPa for both the disposable and reusable housings.

**Fig. 5:**
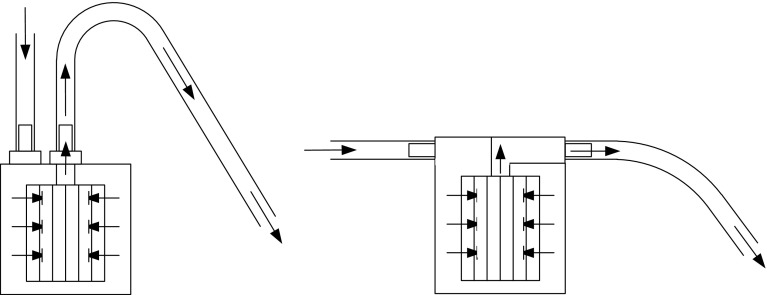
Schematic of filter housing. **a** Disposable housing: flow enters the filter housing, passes through the filter, and then exits the filter housing on a vertical plane. The semi-flexible tubing creates a 0.2-m lost head from the flow exit. **b** Reusable housing: flow enters the filter housing, passes through the filter, and then exits the filter housing on a flat horizontal plane

The extra time required for the disposable housing when using passive filtration is due to its shape (Fig. 5a) as compared to that of the reusable housing (Fig. 5b). For the disposable housing, the inlet and outlet are located on the top of the unit, and the outlet tubing has an inverted “U” shape where the fluid exits the system. This “U” shape reduces the hydrostatic head for gravitational flow by 0.2 m above the filter housing unit and therefore decreases the pressure available to drive filtration. Also, there is insufficient pressure to initially prime the system to allow flow, requiring users to manually wet out the system by “milking,” or squeezing the tubing and increasing the time required to filter the sample. By comparison, the inlet and outlet on the reusable housing are located on the sides of the filter housing lid, increasing the potential hydrostatic head compared to that seen in the disposable housing. The added pressure from this orientation is substantial enough to expel air from the housing and create a siphon effect with minimal user effort. Based on time to filter in the field, it was demonstrated that effective filtration is orientation independent and the reusable housing does not require a stand for use on uneven ground (data not shown).

**Fig. 6:**
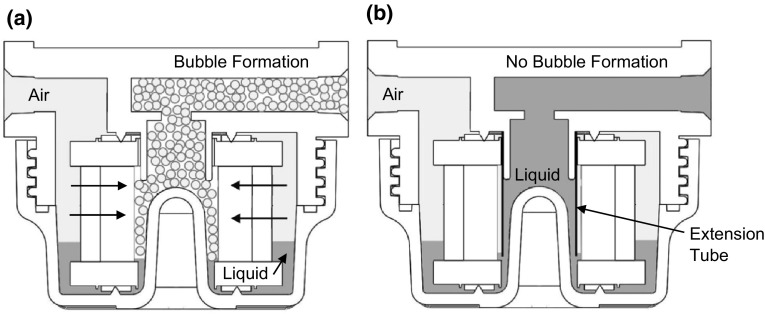
Liquid and air flow in the reusable filter housing. **a** Foam/bubble formation caused by the introduction of air into the liquid phase. **b** Outlet extension tube forces the formation of an air barrier, which reduces foam formation

After field evaluation, the reusable housing was yet further modified to improve its efficiency during the elution process. One major concern with the original design was the formation of air bubbles in the filter housing when the liquid level on the inner side of the filter was higher than the liquid level outside of the filter during the elution procedure (Fig. 6a). This foam was generated by the high-velocity air flow that was pumped through the elution device in order to collect the sample from the filter. The foam persisted in the filter housing and the elution tubing after the elution procedure was finished, decreasing the recovered sample volume and potentially aerosolizing pathogens (Walls et al. 2014). To reduce foam formation during the elution procedure, the filter holder was modified by inserting an extension tube to the suction port (outlet) of the filter housing (Fig. 6b). This repositioning of the suction port near the bottom of the filter housing (Fig. 6b) enables collection of the liquid at the bottom, limiting the air entrainment into the liquid sample. In this case, the liquid level on the outer side of the filter element is always higher than that on the inner side of the filter, establishing an air pocket between the suction location and the filter surface during elution and minimizing the mixing interface between the aqueous and gaseous phases. Therefore, air passing through the filter has no opportunity to mix with liquid directly, limiting the formation of air bubbles and subsequent pathogen aerosolization.

The addition of the extension tube in the filter housing further decreased the dead volume in the reusable housing (Fig. 6b). During elution, the liquid is extracted from the bottom of the filter housing to the outlet, reducing the amount of eluate left in the housing after the elution procedure from 20 to 7 mL.

#### Elution Device Design

In the initial work, a peristaltic pump was used for elution of the samples from the ViroCap filter. While functional, the pump did not enclose the system, which was a safety concern as there was the potential for aerosolization of biologicals from the sample and cross-contamination due to the system not being fully enclosed (Walls et al. 2014). Also, peristaltic pumps are expensive, not easily transferable internationally due to electrical socket and voltage requirements, and complicated to fix with internal and electronic components. Therefore, an elution apparatus was developed to replace the peristaltic pump and for deployment of the self-contained unit to international collaborators. This apparatus does not use electrical power, has a compact footprint (0.093 m^2^), and is compatible with autoclave systems and biosafety cabinets available at the international collaborators’ facilities. In combination with the modified filter housing, the elution device reduced the potential for cross-contamination and increased technician safety, while maintaining similar elution times. A low-cost ($20–$40) manual bilge pump created a vacuum, which exhibited low resistance and did not require significant force to perform the elution procedure. This Alfa Marine pump is not specific for the elution apparatus and so pumps from different manufacturers are compatible with the system, making replacement simple if needed. Use of the system was intuitive and required minimal training (typically less than 30 min). In addition, the elution device encloses the system outlet to reduce the possibility of cross-contamination in the laboratory. The elution device collected the sample into an enclosed sample collection cup; in combination with the optimized filter housing, the possibility of escape of pathogen-containing aerosols from the system was removed (Fig. 3d). If a small amount of bubbles or foam is present in the collection after the elution procedure, the user can wait less than 5 min for the foam to settle in the enclosed container before replacing the ported lid of the elution apparatus with the solid lid for storage and transport of the sample. All components that come in contact with the samples are autoclavable. Finally, the active personnel time required for a single elution using this pump (13 min, *n* = 6) was similar to that using the peristaltic pump (13 min, *n* = 6).

### Virus Recovery Efficiency and Concentration Factor of the BMFS

In addition to modifying the BMFS design to improve usability and performance, the sensitivity of the BMFS was also determined. PV1 recovery measured 39.6 ± 9.6% (*n* = 18) for BMFS samples eluted from ViroCap filters in disposable housings using a single elution (eluate volume 175 mL). With the switch to the reusable housings, the PV1 recovery was 9.7 ± 2.0% (*n* = 21) using a single elution (eluate volume 100 mL). This recovery efficiency was significantly lower than the PV1 recovery with the disposable housing (*p* = 3.9 × 10^−6^, Student’s *t* test). Due to the PV1 recovery decrease and a decrease in the eluate pH, the elution protocol was modified. The new double elution method included two elution steps, each with a hold time of 15 min rather than 30 min and an eluent volume of 100 mL. Using this double elution (total eluate volume 200 mL) and the reusable housings, the PV1 recovery measured 33.1 ± 8.8% (*n* = 11). The PV1 recovery efficiency from the reusable housing using a double elution was not statistically different than the recovery efficiency from the disposable housing using a single elution (*p* = 0.33, Student’s *t* test).

The concentration factor and effective volume assayed for BMFS samples vary by sample volume and type (Table 3). The BMFS can concentrate 3–10 L of wastewater and wastewater-impacted surface waters to 10 mL, depending on the water source (Fagnant et al. 2014). The grab sampling method recommended for polioviruses by the WHO concentrates 500 mL to a final volume of 10 mL using the polyethylene glycol/dextran concentration method (World Health Organization 2015). Therefore, in its current form the BMFS results in an increase of 5–16.7 times the effective volume assayed compared to volumes assayed for 500-mL two-phase grab samples (Table 3). Hence, the increase in effective assay volume may ultimately reduce the likelihood of false-negative samples, compared to samples processed by the WHO algorithm.

**Table 3:** Concentration factors and effective volume plated achieved by use of the BMFS ViroCap reusable filter housing (RH) and disposable filter housing (DH) with two volumes filtered (10 and 3 L) compared to a 0.5-L two-phase grab sample with 10 mL final concentrate volume

Sample type	Volume filtered (*v*_f_, L)	Volume after primary concentration (*v*_1_, mL)	Volume after secondary concentration (*v*_2_, mL)	Volume assayed (*v*_a_, mL)	Primary concentration factor (*c*_1_ = *v*_f_/*v*_1_)	Secondary concentration factor (*c*_2_ = *v*_f_/*v*_2_)	Effective volume assayed (*v*_e_ = *v*_a_ × *c*_2_, mL)
BMFS with RH	10	200^a^	10	3	50^b^	1000	3000
	3	200^a^	10	3	15^c^	300	900
BMFS with DH	10	175	10	3	57.1	1000	3000
	3	175	10	3	17.1	300	900
Two-phase grab sample^d^	0.5	10	N/A	3	50	N/A	150^e^

^a^Volume after double elution. When using a single elution, volume after primary concentration would be 100 mL. Regardless of volume after primary concentration, the volume after secondary concentration is 10 mL

^b^Primary concentration factor after double elution. When using a single elution, primary concentration factor is 100 mL

^c^Primary concentration factor after double elution. When using a single elution, primary concentration factor is 30 mL

^d^Grab sampling method followed by two-phase concentration method recommended for poliovirus sampling by the World Health Organization (WHO)

^e^ *v*_e_ = *v*_a_ × *c*_1_, as no secondary concentration is performed

**Fig. 7:**
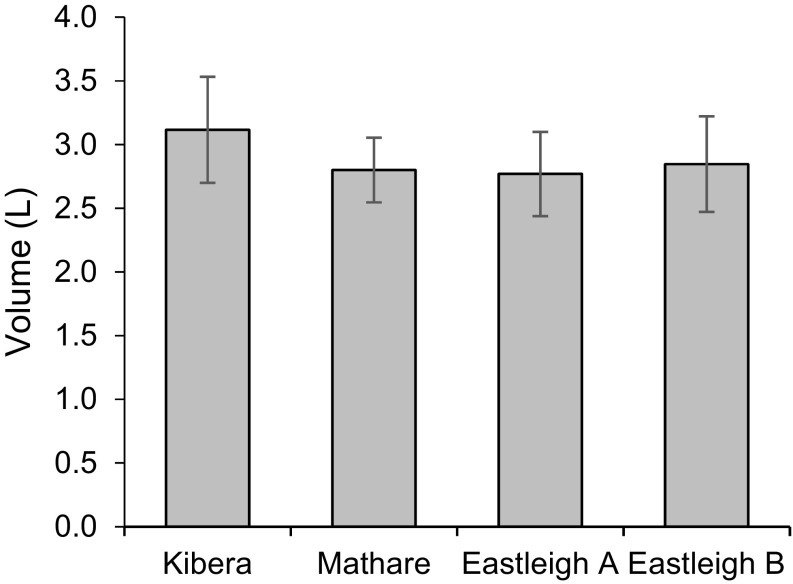
Average volumes filtered through ViroCap disposable housings at sites in Nairobi. For Kibera, Eastleigh A, and Eastleigh B sites, *n* = 13. For the Mathare site, *n* = 15. *Error bars* represent standard deviation

Based on the volumes filtered during the field evaluation, approximately five times the effective volume was assayed with the BMFS compared to the effective volume assayed with the two-phase grab sample method. An average of 2.9 ± 0.4 L was filtered per sample (*n* = 54) (Fig. 7) using the disposable housings and the 6-L third-generation collection bag design at the four sites in Nairobi. Similar volumes were filtered volumes when using the reusable housing.

This compact and transportable field sampling system for enteric viruses requires no external power source, either in the field or in the laboratory. Transitioning from laboratory samples to field samples presented new challenges with water matrix effects, making field testing vital during kit development for troubleshooting these practical challenges to maximize concentrated volumes. Ultimately, use of the BMFS routinely achieved a concentration factor five times greater than that of the currently recommended 0.5-L grab samples processed by the WHO algorithm. With multiple product design iterations, the BMFS now allows for larger filtered volumes in the field, eases sample processing in the laboratory, and may ultimately reduce the likelihood of false-negative samples compared to samples processed by the WHO algorithm, making it a valuable sampling system for surveillance of poliovirus and other enteroviruses in wastewater and wastewater-impacted surface waters.
